# OA01 An innovative model for chronic pain management at the Evelina

**DOI:** 10.1093/rap/rkac066.001

**Published:** 2022-09-28

**Authors:** Megan Viegas, Glenda Dalton, Nick Wilkinson

**Affiliations:** Paediatric Chronic Pain, Evelina Children's Hospital, London, United Kingdom; Paediatric Chronic Pain, Evelina Children's Hospital, London, United Kingdom; Paediatric Chronic Pain, Evelina Children's Hospital, London, United Kingdom

## Abstract

**Introduction/Background:**

Chronic pain is persistent pain lasting more than three months, impacting many aspects of a person’s life. 20-30% of UK adolescents experience chronic pain (although studies vary greatly).

The previous pathway through our service was unclear, which meant young people were not accessing all available resources or being seen promptly.

We aimed to create a model that optimised available resources, including local networks and ensured equitable access to our service across the region. We wanted to target critical barriers such as mental health, school attendance and sleep, and delivering asset-based care.

**Description/Method:**

We followed the “plan, do, study, act” PDSA cycle for this quality improvement project (QIP).

The initial evaluation involved multidisciplinary team (MDT) meetings, site visits, and the MDT experience using other models of care. Patient cases were reviewed for weaknesses in existing models. Discussions highlighted the need to match resources to needs instead of a “one-size-fits-all” approach.

Changes include creating our own “model” for Chronic Pain, introducing a specialist physiotherapy Triage Clinic to the pathway, converting our Pain Education Workshop (PEW) to virtual and utilising local resources by expanding our network.

PEW seeks to empower patients and families to understand their pain. It is also offered to local healthcare professionals to increase confidence in supporting patients.

We adapted the Thrive Mental Health Model to create a Triaged Model for Chronic Pain. We needed to streamline the service, as some patients became lost within the original pathway, not attending PEW and being on long waiting lists. We needed to manage difficulties faced with an increased patient cohort and case complexity, including mental health. Feedback demonstrated some patients found it difficult coming to MDT appointments without knowing who would be present. Triage Clinic helped to mitigate this.

Young people with a lower level of need can be assessed virtually, have symptoms validated, receive advice and be discharged to appropriate care. Those with pain not managed by this reassurance are assessed within our service including, One-Stop-Shop, Adolescent Clinic for Rheumatology patients and Biomechanical clinic. Those requiring more support are referred to the MDT for intervention and education for serial day-case rehabilitation or more intensive intervention (PRIME). Ongoing challenges, such as severe mental health difficulties, mean some may not be ready to access support. We liaise with local services such as CAMHS, and patients may be reintroduced to the team later if appropriate.

**Discussion/Results:**

Our model showed improved outcomes compared to our previous service. We looked at data from 2018-2019 and 2021, removing the years involving the initial waves of the pandemic as the service adapted to the changes this brought.

The physiotherapy Triage Clinic utilised our new Triage Model for the service. Following this we can discharge a portion of our patients to other services, some remain under the physiotherapy team, and the majority are seen in the Chronic Pain MDT clinic. It has been an opportunity, on initial consultation, to engage YP and their families and validate their pain.

22% of our patients had full school attendance on their initial review by our team. The average was a 59% attendance. There was an increase in 83% of those with initially reduced attendance following intervention with the chronic pain team. On average, attendance went from 44% to 71%.

We saw a rise in the number of local services involved with young people under our care. Other services involved safeguarding teams, allied healthcare professionals and CAMHS teams. In 2018 on average, 1.4 services were involved; this increased to 1.8 in 2019 and 2.4 in 2021. This may be due to increased complexity of the cases we see or due to more local support requirements.

Our plans following the MDT clinic increasingly show more specific goals and activities of daily living targets year on year, from 53% in 2018 to 80% having this as part of their plan in 2021.

There have been regional development over the past three years of the service. We now have over 100 staff, including 43 local physiotherapists working collaboratively with our team in supporting the young people and their parents in our care. The Evelina team aims to lead regional meetings and communities of practice.

**Key learning points/Conclusion:**

With the limited available resources, our team is continuing to work towards managing a complex cohort of patients successfully. We were shortlisted for a British Society of Rheumatology Award for Best Practice following the changes made within our department.

This QIP and our new model can be adapted to many other specialities and teams. Changes we have made are especially relevant following the impacts of the Covid-19 pandemic and continued pressure with reduced resources and increased demand.

The virtual PEW allows for increased access for young people that previously struggled to attend face-to-face workshops in a timely fashion. The young people and their families benefit from improving their understanding of their pain and learning new strategies to manage it. Some patients find the virtual setting more practical as they miss less school and do not have to pay for costly transport to attend. Others miss the face-to-face interaction with peers who have similar difficulties. Both individual peer support and expert patients are areas we would like to explore as a service allowing the ripple effect of our model to continue.

While improving the efficiency of some aspects of our service, we know that a further challenge is managing other ‘bottle necks’ created.

From this conference, we hope our new model will be critically appraised and answer questions from our peers in other services. We look forward to learning from the experience of our colleagues who also work with this often complex cohort of patients.

